# Supplementary material for: Benchmarking scientific machine-learning approaches for flow prediction around complex geometries
Source: Commun Eng. 2025 Oct 31;4:182. doi: 10.1038/s44172-025-00513-3 (PMC12578797; doi:10.1038/s44172-025-00513-3)
Supplement: Supplementary file 2 — Supplemenatary Material [file 44172_2025_513_MOESM2_ESM.pdf]

Benchmarking Scientific Machine-Learning Approaches for Flow Prediction Around Complex Geometries

Ali Rabeh[2], Ethan Herron[2], Aditya Balu, Soumik Sarkar, Chinmay Hegde, Adarsh Krishnamurthy[1], Baskar Ganapathysubramanian[1]

Corresponding authors: adarsh@iastate.edu, baskarg@iastate.edu.
† Equal contribution.
AR, EH, AB, SS, AK, and BG are with Iowa State University, Ames, IA, USA.
CH is with NYU Tandon School of Engineering, New York, NY, USA.

Supplementary Material

Supplementary Methods

Data Sufficiency

In this appendix, we explore the data sufficiency requirements for accurate performance of scientific machine learning (SciML) models across varying conditions. We assess model performance on subsets of one-third and one-tenth of the original data using the signed distance field or the binary mask geometry representation. Tables S.1 through S.8 summarize the mean squared errors (MSE) across two difficulty levels, random and exrapolatory, allowing for a detailed comparison of how reduced data availability impacts model accuracy.

Table S.1: *The score of SciML models trained on a subset of half of the dataset using the signed distance field at two different difficulty levels (random and extrapolatory). In this table, M1 denotes the global accuracy ($SDF \geq 0$), M2 denotes the boundary layer accuracy ($0 \leq SDF \leq 0.2$), and M3 denotes the physical consistency using the L_2-norm of the momentum residuals. All errors are reported on the testing dataset.*

	Random			Extrapolatory		
**Model**	**M1**	**M2**	**M3**	**M1**	**M2**	**M3**
**poseidon-L**	56.4	65.3	24.0	**23.9**	**36.9**	28.8
**poseidon-B**	51.9	62.1	23.9	22.7	35.9	30.4
**poseidon-T**	**58.6**	**65.5**	24.1	21.8	36.4	27.6
**scOT-L**	52.2	60.4	25.3	20.9	34.6	30.4
**scOT-B**	48.6	60.5	23.5	20.3	35.2	31.0
**scOT-T**	56.5	63.9	24.3	22.5	37.1	30.1
**CNO**	36.5	47.2	22.5	21.6	33.9	29.4
**FNO**	37.6	56.2	29.1	15.3	28.9	44.4
**WNO**	24.6	40.0	32.4	12.1	25.8	0.0
**Deeponet**	43.3	50.5	**39.3**	19.6	31.0	**44.7**
**geometric-deeponet**	47.1	56.4	35.2	19.7	33.1	42.4

Bold values indicate the best-performing model for that metric.

Table S.2: *The score of SciML models trained on a subset of half of the dataset using the binary mask at two different difficulty levels (random and extrapolatory). In this table, M1 denotes the global accuracy ($SDF \geq 0$), M2 denotes the boundary layer accuracy ($0 \leq SDF \leq 0.2$), and M3 denotes the physical consistency using the L_2-norm of the momentum residuals. All errors are reported on the testing dataset.*

	Random			Extrapolatory		
**Model**	**M1**	**M2**	**M3**	**M1**	**M2**	**M3**
**poseidon-L**	63.4	72.3	24.6	26.1	**41.0**	15.9
**poseidon-B**	56.4	66.5	24.6	21.1	33.6	31.8
**poseidon-T**	**65.7**	**73.1**	24.6	**26.8**	39.8	30.4
**scOT-L**	62.7	70.9	24.5	20.2	33.7	32.4
**scOT-B**	57.0	67.4	24.1	20.0	32.9	33.2
**scOT-T**	63.7	70.7	24.5	20.5	33.5	33.6
**CNO**	40.0	50.4	26.7	22.6	35.1	32.0
**FNO**	34.1	55.5	30.5	18.5	33.4	**39.1**
**WNO**	24.3	38.8	29.8	13.2	27.4	0.0
**Deeponet**	43.9	53.7	32.6	21.3	32.9	37.1
**geometric-deeponet**	45.2	54.0	**32.9**	19.7	33.3	38.6

Bold values indicate the best-performing model for that metric.

Table S.3: *The score of SciML models trained on a subset of one-third of the dataset using the signed distance field at two different difficulty levels (random and extrapolatory). In this table, M1 denotes the global accuracy ($SDF \geq 0$), M2 denotes the boundary layer accuracy ($0 \leq SDF \leq 0.2$), and M3 denotes the physical consistency using the L_2-norm of the momentum residuals. All errors are reported on the testing dataset.*

	**Random**			**Extrapolatory**		
**Model**	**M1**	**M2**	**M3**	**M1**	**M2**	**M3**
**poseidon-L**	53.3	62.0	24.0	**23.9**	36.8	27.2
**poseidon-B**	39.9	48.7	27.0	21.1	34.7	31.1
**poseidon-T**	**56.3**	**63.7**	24.7	20.7	35.9	21.5
**scOT-L**	52.0	60.0	24.8	20.0	34.2	30.5
**scOT-B**	50.8	59.3	24.8	20.4	34.1	31.8
**scOT-T**	54.4	61.6	24.5	22.0	**37.6**	29.1
**CNO**	37.8	45.4	27.5	20.4	30.8	28.8
**FNO**	34.2	53.6	34.5	15.1	28.8	45.8
**WNO**	23.4	38.4	36.2	12.7	26.3	24.4
**Deeponet**	42.4	50.5	**44.9**	22.1	31.4	**51.0**
**geometric-deeponet**	45.2	56.3	34.7	19.5	32.4	42.6

Bold values indicate the best-performing model for that metric.

Table S.4: *The score of SciML models trained on a subset of one-third of the dataset using the binary mask at two different difficulty levels (random and extrapolatory). In this table, M1 denotes the global accuracy ($SDF \geq 0$), M2 denotes the boundary layer accuracy ($0 \leq SDF \leq 0.2$), and M3 denotes the physical consistency using the L_2-norm of the momentum residuals. All errors are reported on the testing dataset.*

	**Random**			**Extrapolatory**		
**Model**	**M1**	**M2**	**M3**	**M1**	**M2**	**M3**
**poseidon-L**	50.7	62.7	21.9	28.2	42.5	27.6
**poseidon-B**	49.1	59.8	24.4	20.8	33.4	32.5
**poseidon-T**	**55.6**	63.7	26.3	**29.3**	**46.8**	23.2
**scOT-L**	54.8	**64.3**	25.2	20.5	33.4	32.3
**scOT-B**	53.1	62.3	24.8	19.7	33.0	33.7
**scOT-T**	52.3	62.7	23.9	20.4	33.1	32.9
**CNO**	42.5	52.5	28.3	18.5	30.6	34.4
**FNO**	32.3	53.8	33.7	17.2	31.6	**43.1**
**WNO**	24.1	39.1	27.3	13.1	26.6	15.3
**Deeponet**	41.2	52.4	**36.4**	20.8	32.6	40.3
**geometric-deeponet**	45.5	54.4	33.7	18.1	31.3	**43.1**

Bold values indicate the best-performing model for that metric.

Table S.5: *The score of SciML models trained on a subset of one-sixth of the dataset using the signed distance field at two different difficulty levels (random and extrapolatory). In this table, M1 denotes the global accuracy ($SDF \geq 0$), M2 denotes the boundary layer accuracy ($0 \leq SDF \leq 0.2$), and M3 denotes the physical consistency using the L_2-norm of the momentum residuals. All errors are reported on the testing dataset.*

	**Random**			**Extrapolatory**		
**Model**	**M1**	**M2**	**M3**	**M1**	**M2**	**M3**
**poseidon-L**	**46.8**	**57.9**	24.5	**27.1**	38.0	24.0
**poseidon-B**	43.6	52.6	25.1	21.4	34.9	31.3
**poseidon-T**	43.5	53.7	25.2	25.7	**42.0**	19.0
**scOT-L**	45.9	54.5	27.1	19.4	33.3	31.7
**scOT-B**	45.7	55.4	24.3	19.3	31.5	33.5
**scOT-T**	45.1	53.7	28.0	19.3	32.6	33.9
**CNO**	36.4	47.3	27.6	20.2	32.3	33.4
**FNO**	32.4	50.5	37.7	15.7	29.7	46.1
**WNO**	22.2	38.6	0.0	11.9	25.9	0.0
**Deeponet**	37.7	47.9	**50.3**	17.2	28.8	**58.9**
**geometric-deeponet**	40.3	52.9	43.7	19.5	32.6	43.6

Bold values indicate the best-performing model for that metric.

Table S.6: *The score of SciML models trained on a subset of one-sixth of the dataset using the binary mask at two different difficulty levels (random and extrapolatory). In this table, M1 denotes the global accuracy ($SDF \geq 0$), M2 denotes the boundary layer accuracy ($0 \leq SDF \leq 0.2$), and M3 denotes the physical consistency using the L_2-norm of the momentum residuals. All errors are reported on the testing dataset.*

	**Random**			**Extrapolatory**		
**Model**	**M1**	**M2**	**M3**	**M1**	**M2**	**M3**
**poseidon-L**	50.4	60.1	25.0	**34.5**	**50.6**	12.6
**poseidon-B**	44.8	54.1	25.3	26.3	39.9	25.1
**poseidon-T**	**54.7**	**62.9**	26.3	30.2	48.0	27.9
**scOT-L**	51.3	60.1	24.5	17.5	32.4	28.0
**scOT-B**	47.8	58.0	23.5	20.4	32.7	33.8
**scOT-T**	45.1	55.7	27.2	22.7	36.5	29.8
**CNO**	32.7	44.9	26.9	24.5	36.2	28.6
**FNO**	30.9	51.2	35.9	16.4	31.1	45.0
**WNO**	22.4	38.4	0.0	11.5	26.2	0.0
**Deeponet**	39.2	52.0	37.0	17.6	29.9	**49.8**
**geometric-deeponet**	40.0	51.2	**37.7**	18.9	32.1	41.7

Bold values indicate the best-performing model for that metric.

Table S.7: *The score of SciML models trained on a subset of one-tenth of the dataset using the signed distance field at two different difficulty levels (random and extrapolatory). In this table, M1 denotes the global accuracy ($SDF \geq 0$), M2 denotes the boundary layer accuracy ($0 \leq SDF \leq 0.2$), and M3 denotes the physical consistency using the L_2-norm of the momentum residuals. All errors are reported on the testing dataset.*

	Random			Extrapolatory		
**Model**	**M1**	**M2**	**M3**	**M1**	**M2**	**M3**
**poseidon-L**	**45.0**	**56.6**	22.0	**28.7**	**39.5**	16.5
**poseidon-B**	44.7	54.2	23.0	25.9	38.5	26.9
**poseidon-T**	43.8	54.4	26.4	24.5	39.4	22.8
**scOT-L**	42.9	52.2	28.6	18.3	33.6	27.8
**scOT-B**	43.1	53.6	24.8	19.5	32.8	32.0
**scOT-T**	42.7	52.2	30.4	19.8	33.9	33.9
**CNO**	32.7	44.6	25.0	21.9	34.6	32.3
**FNO**	32.0	51.9	35.6	14.3	27.9	51.4
**WNO**	17.9	37.5	0.0	12.1	25.6	0.0
**Deeponet**	35.0	46.4	**51.8**	18.2	29.4	**57.0**
**geometric-deeponet**	34.9	47.9	44.5	18.6	32.1	48.7

Bold values indicate the best-performing model for that metric.

Table S.8: *The score of SciML models trained on a subset of one-tenth of the dataset using the binary mask at two different difficulty levels (random and extrapolatory). In this table, M1 denotes the global accuracy ($SDF \geq 0$), M2 denotes the boundary layer accuracy ($0 \leq SDF \leq 0.2$), and M3 denotes the physical consistency using the L_2-norm of the momentum residuals. All errors are reported on the testing dataset.*

	Random			Extrapolatory		
**Model**	**M1**	**M2**	**M3**	**M1**	**M2**	**M3**
**poseidon-L**	45.9	57.5	21.8	**30.2**	47.0	13.6
**poseidon-B**	**50.9**	60.5	25.0	26.9	42.7	23.3
**poseidon-T**	50.8	**60.9**	30.3	29.6	**47.3**	27.1
**scOT-L**	44.2	54.4	29.6	18.0	32.6	26.2
**scOT-B**	46.4	56.0	27.1	19.8	33.8	30.3
**scOT-T**	38.7	50.7	29.5	18.9	31.7	35.5
**CNO**	30.6	42.5	40.3	18.4	30.3	40.8
**FNO**	29.8	50.9	**36.7**	14.6	28.4	51.1
**WNO**	21.4	37.5	0.0	10.7	25.5	0.0
**Deeponet**	37.1	52.0	36.1	16.2	28.5	**53.7**
**geometric-deeponet**	38.6	53.0	36.0	18.8	32.1	42.2

Bold values indicate the best-performing model for that metric.

Field Predictions

In Figure S.1, Figure S.2, and Figure S.3, we present field velocity predictions in the y-direction v for a representative sample using scientific machine learning models (CNO, geometric-DeepONet, Poseidon-T).

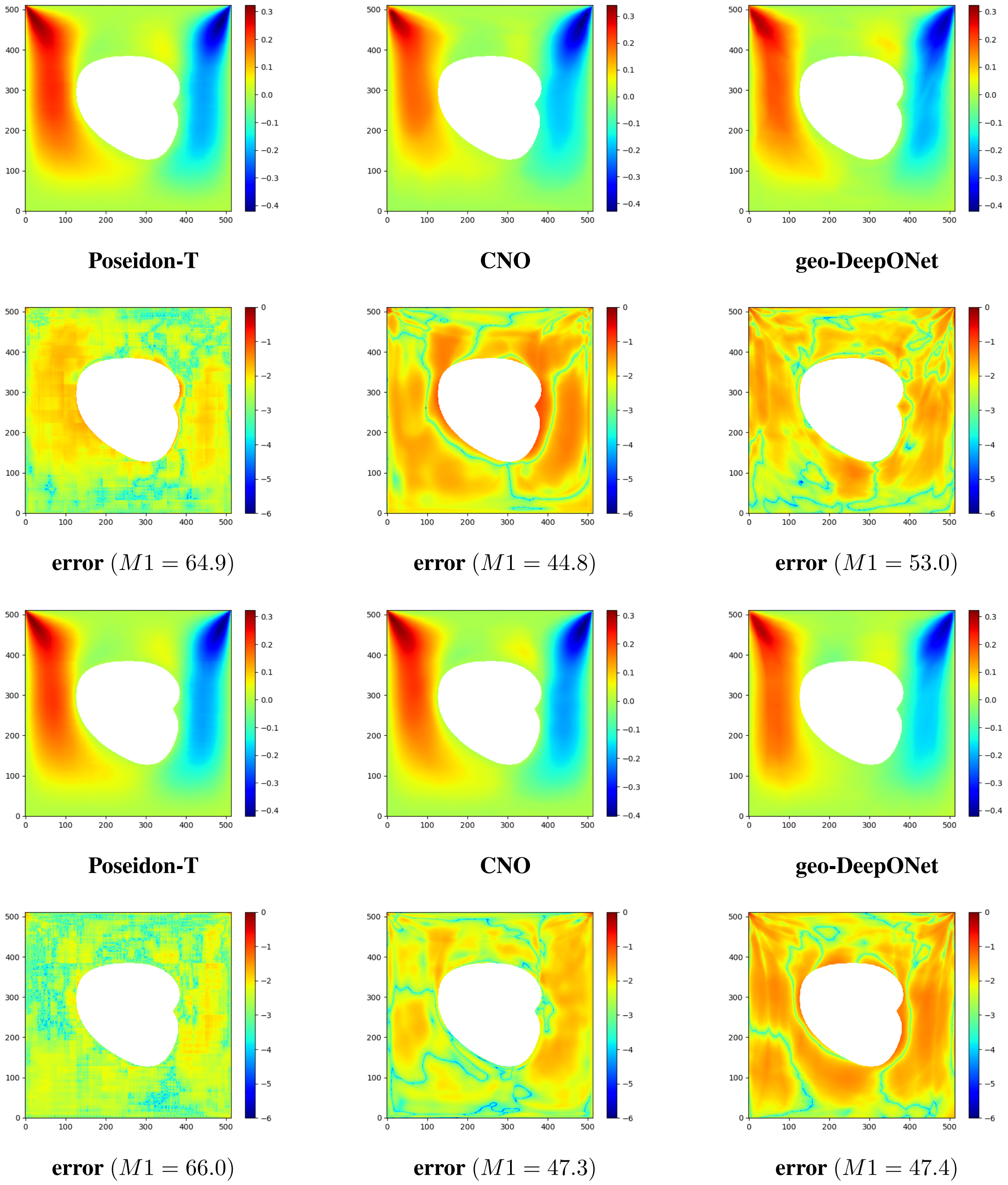

Figure S.1: *Comparison of model predictions and log-scale error distributions using SDF and mask geometry representations. The first row shows predictions based on the SDF representation, followed by the second row displaying the corresponding log-scale error maps, with each error image annotated with its global accuracy score ($M1$). The third and fourth rows present predictions and log-scale error maps using the mask representation.*

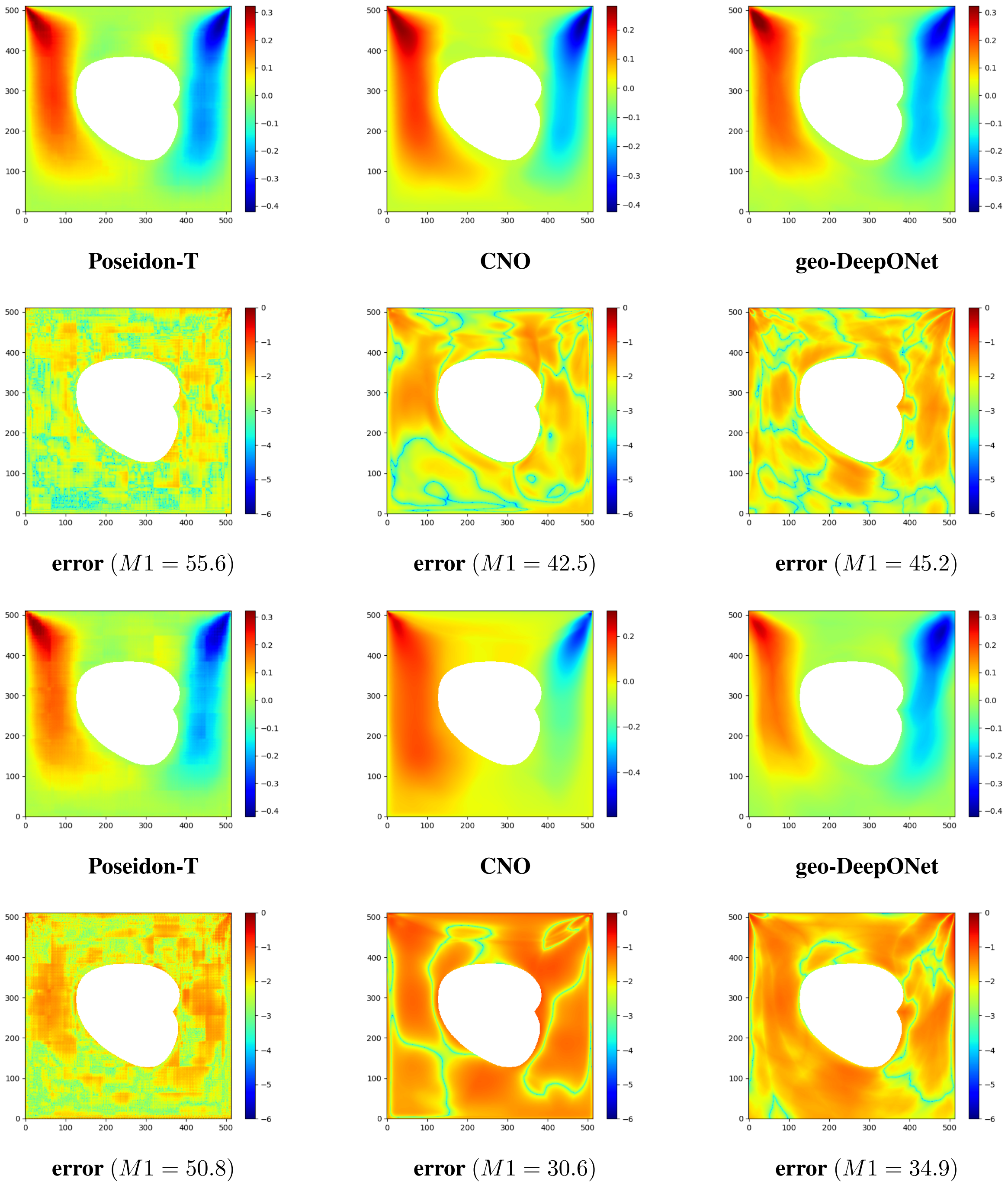

Figure S.2: *Comparison of model predictions and log-scale error distributions using training sample sizes of 800 and 240, highlighting data sufficiency. The first row shows predictions using a training sample size of 800, featuring Geometric-DeepONet with the Signed Distance Field (SDF) representation and CNO and Poseidon-T using the binary mask, followed by the second row displaying corresponding log-scale error maps, with each error image annotated with its global accuracy score ($M1$). The third and fourth rows present the corresponding predictions and log-scale error maps using a sample size of 240.*

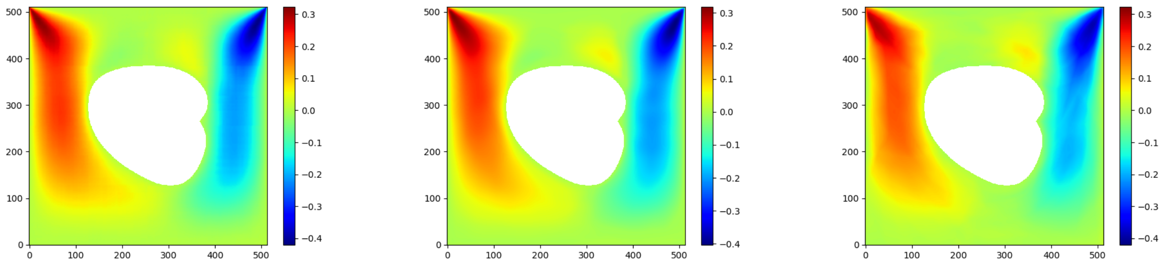

Poseidon-T **CNO** **geo-DeepONet**

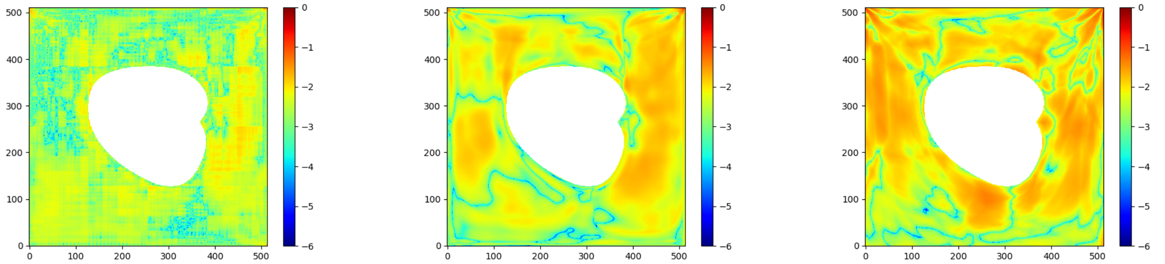

error $(M1 = 66.0)$ **error** $(M1 = 47.3)$ **error** $(M1 = 53.0)$

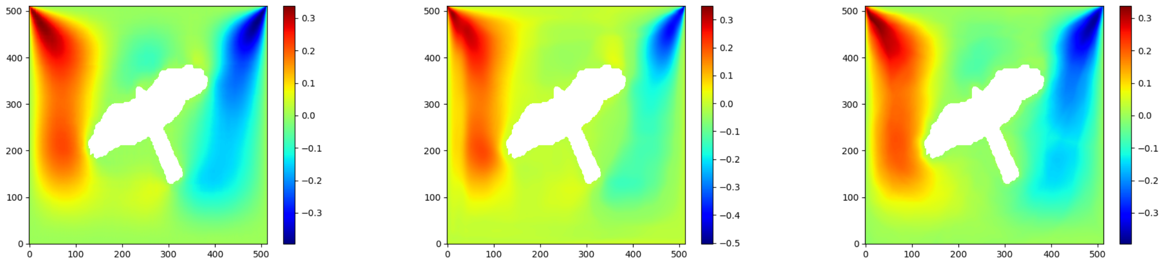

Poseidon-T **CNO** **geo-DeepONet**

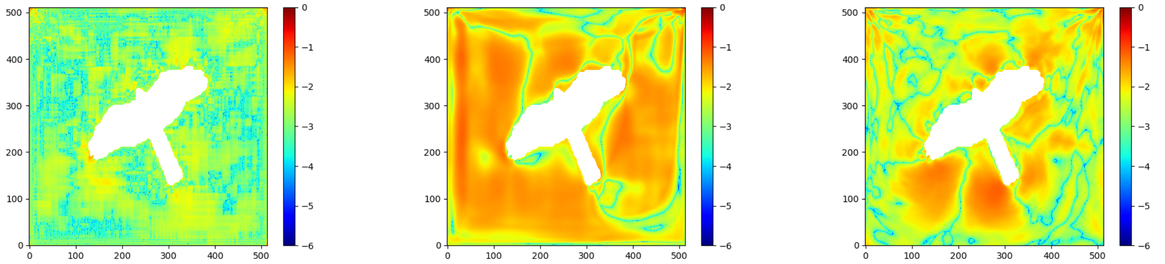

error $(M1 = 23.4)$ **error** $(M1 = 26.5)$ **error** $(M1 = 25.0)$

Figure S.3: *Comparison of model predictions and log-scale error distributions for random train/test splitting and extrapolatory train/test splitting, highlighting the impact of different data splits. The first row shows predictions using the random split, featuring Geometric-DeepONet with the Signed Distance Field (SDF) representation and CNO and Poseidon-T using the binary mask, followed by the second row displaying corresponding log-scale error maps, with each error image annotated with its global accuracy score (*$M1$*). The third and fourth rows present the corresponding predictions and log-scale error maps using the extrapolatory split.*

Residual Calculation

The finite element mesh, denoted by $\mathcal{K}_h$, defines the computational discretization of the domain Ω. Integrations over Ω are computed by integrating within each finite element e and summing over all elements in $\mathcal{K}_h$. For instance, the L_2-norm of the velocity component u over Ω is defined as:

$$\|u\|_{L_2(\Omega)} = \left(\int_\Omega u^2 \, d\Omega\right)^{1/2} = \left(\sum_{e \in \mathcal{K}_h} \int_e u^2 \, de\right)^{1/2}$$

Similarly, for the Navier-Stokes momentum residuals, we compute the element-wise residuals. These residuals, denoted by r_x and r_y, represent the momentum conservation equations in the x- and y-directions, respectively.

$$r_\text{x} = \frac{\partial u_x}{\partial t} + u \cdot \nabla u_x - \eta \nabla^2 u_x + \frac{\partial p}{\partial x}$$
$$r_\text{y} = \frac{\partial u_y}{\partial t} + u \cdot \nabla u_y - \eta \nabla^2 u_y + \frac{\partial p}{\partial y}$$

These element-wise residuals are calculated as follows:

$$\|r_\text{x}\|_{L_2(e)} = \left(\int_e r_\text{x}^2 \, de\right)^{1/2}$$

$$\|r_\text{y}\|_{L_2(e)} = \left(\int_e r_\text{y}^2 \, de\right)^{1/2}$$

The total residual r_Total (denoted $M3$) is then obtained by adding the contributions of each element:

$$r_\text{Total} = \sum_{e \in \mathcal{K}_h} \left(\|r_\text{x}\|^2_{L_2(e)} + \|r_\text{y}\|^2_{L_2(e)}\right)$$

In addition to the residuals, we compute the errors in the spatial derivatives of the velocity components, u and v, by comparing the predicted derivatives with their ground truth gradient values. These errors, denoted u'_error and v'_error, respectively, are defined as:

$$u'_\text{error} = \left(\frac{\partial u}{\partial x} - \frac{\partial u_\text{true}}{\partial x}\right)^2 + \left(\frac{\partial u}{\partial y} - \frac{\partial u_\text{true}}{\partial y}\right)^2$$
$$v'_\text{error} = \left(\frac{\partial v}{\partial x} - \frac{\partial v_\text{true}}{\partial x}\right)^2 + \left(\frac{\partial v}{\partial y} - \frac{\partial v_\text{true}}{\partial y}\right)^2$$

These errors are integrated element-wise to calculate their L_2-norms over each element e:

$$\|u'_\text{error}\|_{L_2(e)} = \left(\int_e u'_\text{error} \, de\right)^{1/2}, \quad \|v'_\text{error}\|_{L_2(e)} = \left(\int_e v'_\text{error} \, de\right)^{1/2}$$

The residual metric assesses the accuracy of the velocity and pressure fields within each element and measures the adherence to momentum conservation throughout the domain, while the derivative error quantifies discrepancies in velocity gradients compared to ground truth gradient values.

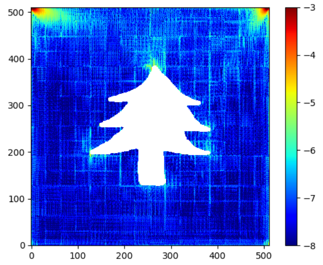

Poseidon-T using SDF (r_x)

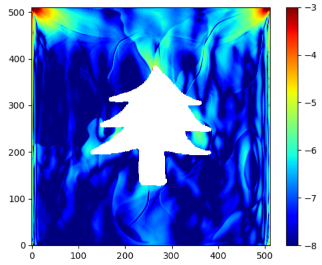

CNO using SDF (r_x)

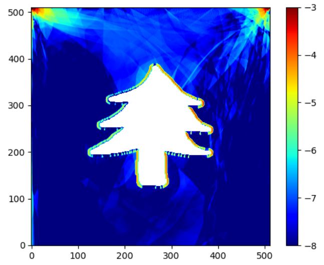

DeepONet using SDF (r_x)

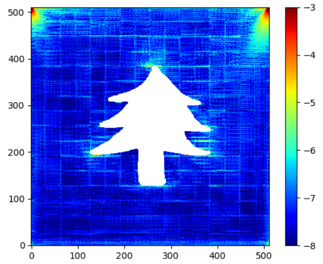

Poseidon-T using SDF (r_y)

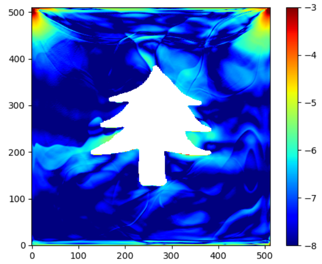

CNO using SDF (r_y)

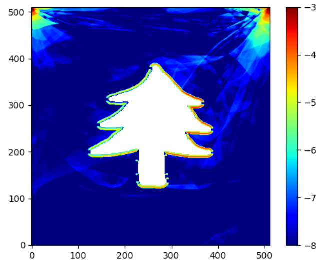

DeepONet using SDF (r_y)

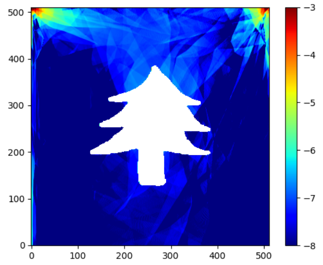

Poseidon-T using mask (r_x)

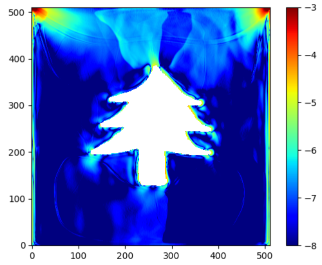

CNO using mask (r_x)

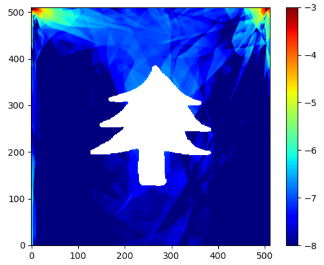

DeepONet using mask (r_x)

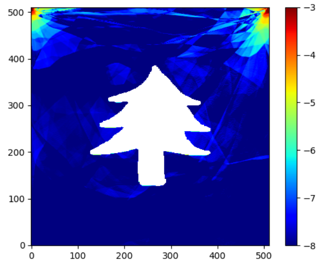

Poseidon-T using mask (r_y)

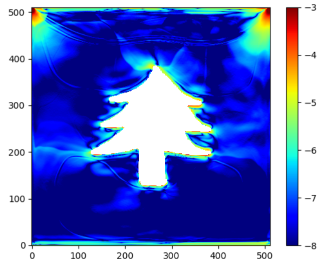

CNO using mask (r_y)

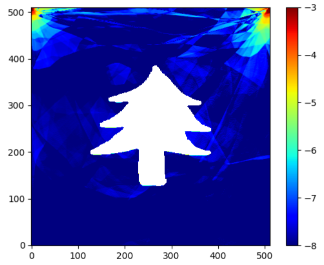

DeepONet using mask (r_y)

Figure S.4: *Comparison of model residual distributions in the x and y directions (r_x, r_y) for a single sample using random train/test splitting, highlighting the impact of different geometry representations. The first two rows show r_x and r_y (respectively) in the log scale using the signed distance field, featuring poseidon-T, CNO, and DeepONet. The third and fourth rows present r_x and r_y (respectively) in the log scale using the binary mask, featuring poseidon-T, CNO, and DeepONet.*

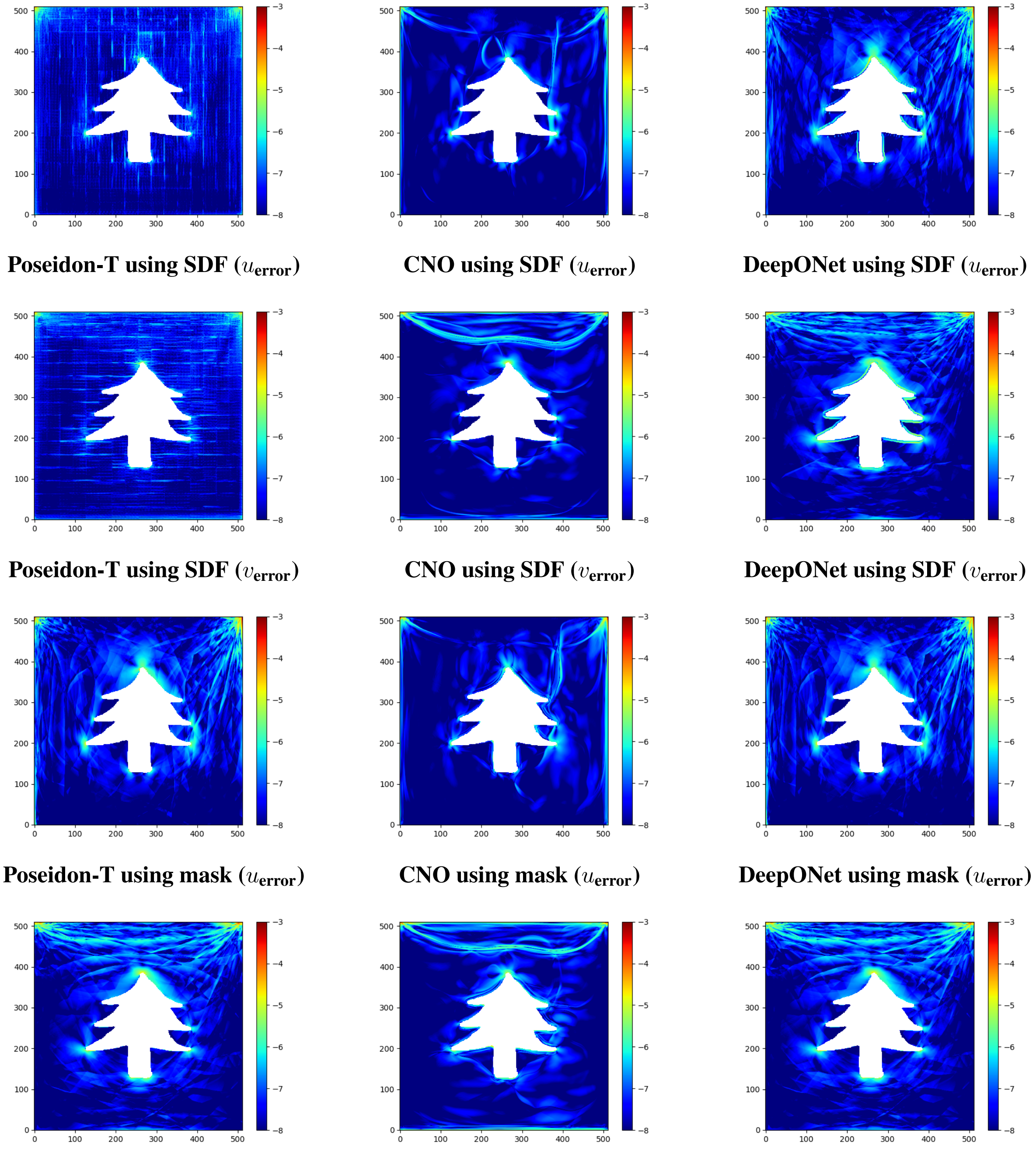

Poseidon-T using SDF (u_{error}) **CNO using SDF (u_{error})** **DeepONet using SDF (u_{error})**

Poseidon-T using SDF (v_{error}) **CNO using SDF (v_{error})** **DeepONet using SDF (v_{error})**

Poseidon-T using mask (u_{error}) **CNO using mask (u_{error})** **DeepONet using mask (u_{error})**

Poseidon-T using mask (v_{error}) **CNO using mask (v_{error})** **DeepONet using mask (v_{error})**

Figure S.5: *Comparison of model velocity gradient error distributions in the x and y directions (u'_{error}, v'_{error}) for a single sample using random train/test splitting, highlighting the impact of different geometry representations. The first two rows show u'_{error} and v'_{error} (respectively) in the log scale using the signed distance field, featuring poseidon-T, CNO, and DeepONet. The third and fourth rows present u'_{error} and v'_{error} (respectively) in the log scale using the binary mask, featuring poseidon-T, CNO, and DeepONet.*

Model Hyperparameters

This section provides a detailed overview of the hyperparameters used for training each of the 11 scientific machine learning (SciML) models. These hyperparameters were selected based on extensive tuning to optimize performance in the validation set (20% of the training data, as described in Validation Dataset and Hyperparameter Tuning) by minimizing the validation loss of the global accuracy metric $M1$.

Convolution Neural Operator

The Convolution Neural Operator (CNO) was trained with the following hyperparameters:

- Number of residual blocks (`N_res`): 4
- Learning rate (`lr`): 0.001
- Number of layers (`n_layers`): 4

Fourier Neural Operator

The Fourier Neural Operator (FNO) was trained with the following hyperparameters:

- Hidden channels (`hidden_channels`): 16
- Learning rate (`lr`): 0.0001
- Number of layers (`n_layers`): 10
- Number of Fourier modes (`n_modes`): [64, 64]
- Projection channels (`projection_channels`): 16

DeepONet and Geometric DeepONet

The hyperparameters for the standard DeepONet and its geometric extension are as follows:

- Branch network layers (`branch_net_layers`): [512, 512, 512]
- Trunk network layers (`trunk_net_layers`): [256, 256, 256]
- Learning rate (`lr`): 0.0001
- Modes (`modes`): 128

Wavelet Neural Operator

The Wavelet Neural Operator (WNO) was trained with the following hyperparameters:

- Input channels (`in_channels`): 4
- Wavelet decomposition level (`level`): 4
- Learning rate (`lr`): 0.001
- Network width (`width`): 64

scOT-T and poseidon-T

The hyperparameters for the tiny versions of scOT and Poseidon are as follows:

- Depths (`depths`): [4, 4, 4, 4]
- Embedding dimension (`embed_dim`): 48
- Learning rate (`lr`): 0.0005

scOT-B and poseidon-B

The hyperparameters for the base versions of scOT and Poseidon are as follows:

- Depths (`depths`): [8, 8, 8, 8]
- Embedding dimension (`embed_dim`): 96
- Learning rate (`lr`): 0.0005

scOT-L and poseidon-L

The hyperparameters for the large versions of scOT and Poseidon are as follows:

- Depths (`depths`): [8, 8, 8, 8]
- Embedding dimension (`embed_dim`): 192
- Learning rate (`lr`): 0.0001

Training and Validation Loss

To further analyze the training performance, the evolution of training and validation loss for four representative models (Poseidon-T, scOT-T, FNO, and DeepONet) are shown in Figure S.6. During training, we reserve 80% of the available training data for training and 20% for validation to monitor model generalization. The data is loaded with shuffling enabled so that the training samples are presented in a random order at each epoch.

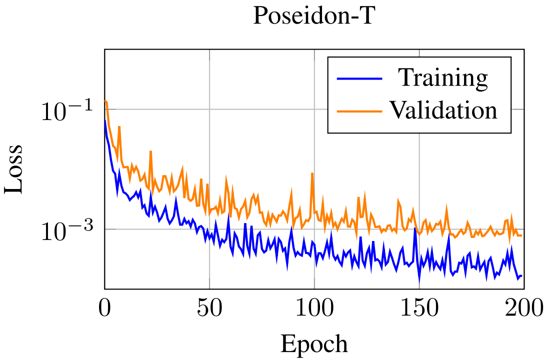

(a) *Poseidon-T*

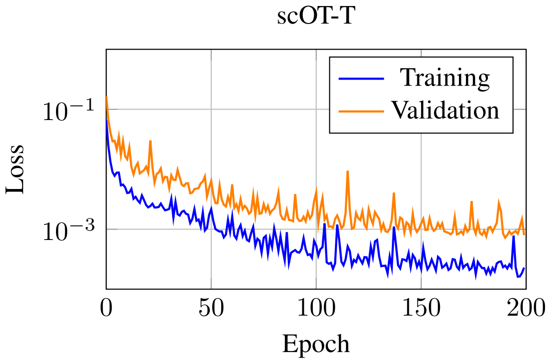

(b) *scOT-T*

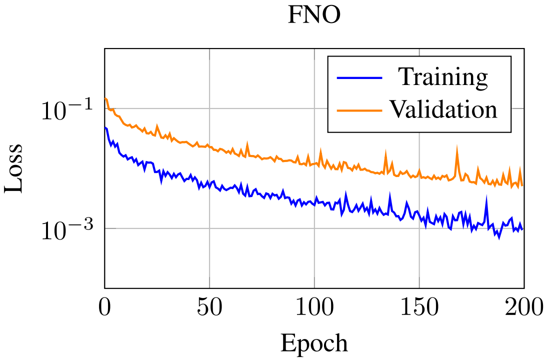

(c) *FNO*

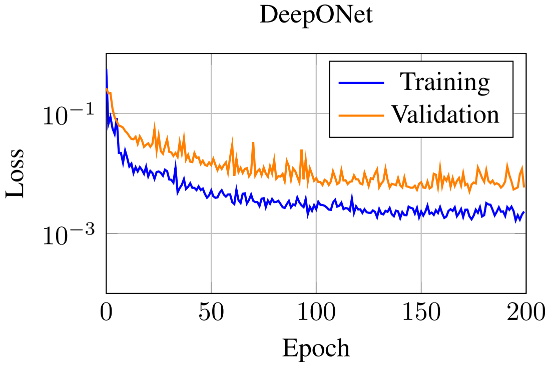

(d) *DeepONet*

Figure S.6: *Training and validation loss (semi-log scale) for four models: Poseidon-T, scOT-T, FNO, and DeepONet.*
